# Allopurinol: Clinical Considerations in the Development and Treatment of Stevens-Johnson Syndrome, Toxic Epidermal Necrolysis, and Other Associated Drug Reactions

**DOI:** 10.7759/cureus.64654

**Published:** 2024-07-16

**Authors:** Kathryn M Dillman, Alison M Hawkins, Amanda R Ragland, Grace C Wester, Driskell R Greene, Giustino Varrassi, Peyton Moore, Raju Behara, Shahab Ahmadzadeh, Harish Siddaiah, Sahar Shekoohi, Alan D Kaye

**Affiliations:** 1 Medicine, Louisiana State University Health Sciences Center New Orleans, New Orleans, USA; 2 Medicine, Louisiana State University Health Sciences Center, Shreveport, USA; 3 Pain Medicine, Paolo Procacci Foundation, Rome, ITA; 4 Anesthesiology, Louisiana State University Health Sciences Center, Shreveport, USA

**Keywords:** gout, immune system, drug reaction, hypersensitivity, oxypurinol, allopurinol, toxic epidermal necrolysis, stevens-johnson syndrome

## Abstract

Allopurinol lowers urate production through the inhibition of xanthine oxidase. It is oxidatively hydroxylated to oxypurinol and is the most prescribed medication for gout treatment. Although it has a beneficial effect in the treatment of this common disease, like many medications, it is also known for having numerous adverse effects. Stevens-Johnson syndrome (SJS) and toxic epidermal necrolysis (TEN), diseases that exist on a spectrum, are two of the most dangerous adverse effects associated with allopurinol use. These immune-mediated disease processes involve almost every organ system. They are essential to recognize as early as possible, as they could potentially be deadly, requiring cessation of the medication with initial signs of rash or other early manifestations of SJS/TEN. One major consideration in the increased risk of allopurinol-mediated or modulated SJS/TEN is the need to have a lower dose in the setting of renal disease. The purpose of this review is not only to examine the involvement of allopurinol in SJS/TEN but also to provide detailed information about the drug, allopurinol, and general features and characteristics of SJS/TEN and other associated drug reactions.

## Introduction and background

Stevens-Johnson syndrome (SJS) and toxic epidermal necrolysis (TEN) are immune-mediated mucocutaneous reactions frequently associated with medications and characterized by blistering lesions, necrosis, and involvement of several organ systems [1]. It has been reported that there are anywhere from 100 to 200 different medications associated with the development of these reactions, and exposure to one of these medications is present in at least half, if not more, of these cases [1,2]. Some common groups of implicated medications include sulfa-containing drugs, antibiotics, anticonvulsants, antineoplastic agents, and allopurinol, which is considered the most common medication associated with SJS/TEN [3-5]. If not medication-related, SJS and TEN can also develop secondary to infections, particularly Mycoplasma pneumoniae, and malignancies, both hematologic and non-hematologic [1]. These reactions are immune-mediated, typically favoring excessive T-cell activation over B cell involvement [6]. Despite the lack of B cell involvement, several cytokines also play a role in these reactions, such as granulysin, perforin, granzyme B, and tumor necrosis factor (TNF). These cytokines are especially important for the apoptotic aspect of the immune reaction [7]. SJS and TEN typically manifest within the first few days of exposure to a particular medication but can occur as late as 28 days post-exposure with a rash, high fevers, malaise, and arthralgias [5,8]. Other common initial symptoms include headaches, pharyngitis, conjunctivitis, and skin lesions [9]. The characteristic skin lesion begins as a plaque, papule, or blister, which becomes superimposed on a target or macular lesion and eventually results in epidermal necrosis [4,10,11]. A diffuse distribution across the skin, including the involvement of mucosal surfaces leading to ophthalmologic and bronchial manifestations, is very characteristic of these reactions and helps to differentiate them from other common skin lesions like erythema multiforme [9]. Other systems involved include the gastrointestinal, renal, hematologic, and endocrine systems [1].

Allopurinol, the most common medication known to cause SJS and TEN, is a xanthine oxidase inhibitor frequently used in the treatment and management of gout and calcium nephrolithiasis and the prevention of tumor lysis syndrome. As a xanthine oxidase inhibitor, it prevents the conversion of hypoxanthine to xanthine and eventually to uric acid in the purine catabolism pathway. Allopurinol, which has a half-life of one to three hours, is metabolized in the liver to oxypurinol, the active metabolite with a half-life of 12-17 hours, and is excreted renally [12]. Thus, in managing gout, calcium nephrolithiasis, or tumor lysis syndrome in those with acute or chronic kidney disease (CKD), the starting dose of allopurinol is significantly reduced [13]. While it is generally regarded as a safe medication, one of the more severe adverse effects is allopurinol hypersensitivity syndrome (AHS), which has a mortality rate of 20-25%. AHS is defined by several clinical features, including vasculitis, hepatocellular injury, acute kidney injury, fever, leukocytosis, eosinophilia, and the topics of this review, both SJS and TEN [14]. While SJS and TEN can occur in the context of AHS, they have also been reported to occur in isolation after exposure to allopurinol [15]. Other adverse effects outside of AHS include acute gouty flares on initiation of treatment, a maculopapular pruritic rash, nausea, diarrhea, transaminitis, elevated serum alkaline phosphatase, leukopenia, thrombocytopenia, hepatoxicity, and subclinical hypothyroidism [16-18].

This review further discusses allopurinol and its clinical utility and treatment recommendations, the pathophysiology and clinical presentation of SJS and TEN, and the relationship of allopurinol to SJS and TEN, augmented by a summary of clinical cases of allopurinol-induced SJS and TEN. Additionally, an in-depth review of treatment options for these disease processes is described. Finally, other allopurinol-mediated and/or modulated drug reactions outside of SJS and TEN are further discussed.

## Review

Allopurinol mechanism of action, treatment indications, and adverse effects

Allopurinol, a xanthine oxidase inhibitor, is frequently used in the management of gout as a urate-lowering medication. Besides gout management, it has otherwise been approved for use in the prevention of both tumor lysis syndrome and recurrent calcium nephrolithiasis [12]. This section aims to explore the role of allopurinol in the treatment of gouty arthritis, its pharmacokinetics and pharmacodynamics, and associated adverse effects, along with the clinical risk factors that may precipitate them.

Acute Gout Treatments

There are various treatment options for gout, depending on its presentation: acute versus chronic. Acute gout is classified as an attack that has a rapid onset (defined as a symptomatic peak of 12-24 hours) and is characterized by excruciating pain in a joint with immense tenderness to touch, typically resolving within one to two weeks [19]. The first-choice drugs for acute gout treatment are non-steroidal anti-inflammatory drugs (NSAIDs), corticosteroids, colchicine, cortisone, and IL-1 antagonists [20]. The three former medications are the most commonly prescribed for acute attacks; however, it is necessary to consider the possible contraindications. For NSAIDs, one must consider renal failure; for corticosteroids, one must consider the production of stomach acid and Cushing’s syndrome; and finally, for colchicine, the cytochrome P (CYP)3A4 mechanism of metabolism must be considered, as well as the gastrointestinal side effects [20].

Chronic Gout Treatments

Chronic gout develops after years of inflammation brought on by repetitive acute attacks. In extensive or repetitive disease courses, one can use chronic gout treatments such as xanthine oxidase inhibitors like allopurinol. One should not begin long-term xanthine oxidase treatment until after the acute attack has resolved, as beginning a long-term treatment could exacerbate the attack [20]. The National Institute for Health and Care Excellence recommends waiting at least two weeks to begin treatment with allopurinol to avoid this exacerbation and allow the patient time to recover and make appropriate decisions regarding their care [21]. The American College of Rheumatology’s guidelines for long-term therapy describe the following criteria: the recommended dosing is initiated at <100 mg/day with up to a maximum daily dose of 800 mg/day until a target serum uric acid level of <6 mg/dL is reached [22]. The dose should be adjusted and lowered based on cases of known renal failure. The patient’s liver function and kidney function should also be monitored with routine laboratory work every few weeks and then every few months once target uric acid levels are achieved [22]. The American College of Rheumatology clinically recommends treatment with allopurinol or other urate-lowering therapy during an acute flare if the decision to begin this therapy is made while an active flare is in progress [22]. The benefits of starting therapy during a flare include high patient motivation and mitigating the risk of a patient not following up for allopurinol initiation [22]. Despite these benefits, the American College of Rheumatology acknowledges concerns about the potential prolongation or worsening of the flare [22].

Pharmacokinetics and pharmacodynamics of allopurinol

Allopurinol is metabolized and oxidized in the liver into its active metabolite, oxipurinol, and then the major mechanism of its elimination is via the excretion of oxipurinol through the kidneys [23]. Oxipurinol has a significantly longer t½ than allopurinol. While the mean t½ for allopurinol after oral administration is approximately 1.2 hours, the t½ for oxipurinol is 23 hours [24]. Allopurinol is mainly cleared via nonrenal mechanisms [23]. Oxipurinol, on the other hand, is mainly cleared. In a healthy individual, the clearance rate for oxipurinol is about 23 mL/min [24]. However, due to its renal clearance mechanism, patients with renal impairment have decreased clearance. This also accounts for differences in oxipurinol clearance in elderly patients with age-related kidney impairment compared to young adults [23]. As for the pharmacodynamics of allopurinol, plasma uric acid concentrations decreased in response to the drug in both elderly and young adult populations [23]. However, elderly subjects appear to have less of a decrease than young patients, indicating age-related differences [23].

Clinical risk factors for adverse effects in allopurinol users

While allopurinol is frequently prescribed in part due to its minimal side effects, it has been shown to result in an array of adverse events. In a study involving 1,934 patients, about 5% experienced adverse events [25]. The most common among these are skin rashes, GI issues, severe cutaneous adverse reactions (SCARs) such as SJS and TEN, AHS, fever, musculoskeletal events, and neurologic events [25]. The medication-associated risk factors included patients taking higher doses of allopurinol and those taking colchicine or statins concurrently with allopurinol [26]. Other studies have indicated that taking thiazide diuretics also puts patients at risk of severe AHS as they alter the sodium and urate processing abilities of the kidney [27]. Additionally, genetics have been found to play a role in severe reactions to allopurinol. The human leukocyte antigen (HLA) B*58:01 allele has been reported as a significant risk factor for adverse events in Chinese (specifically of Han Chinese descent), Japanese, and European populations [28].

Allopurinol hypersensitivity

As mentioned previously, allopurinol use is associated with adverse reactions such as mild skin rash, GI issues, SCAR such as SJS and TEN, AHS, fever, musculoskeletal events, and neurologic events [25]. While AHS is rare, seen in only 0.1% of patients, it can be associated with significant mortality [23]. In Europe and Israel, allopurinol hypersensitivity is the most common cause of SJS and TEN and the second most common cause of drug reactions with eosinophilia and systemic symptoms [23]. SJS and TEN can be life-threatening type IV hypersensitivity reactions that typically present within one month of beginning treatment [1]. The presentation normally begins with common fever symptoms, such as headache, arthralgias, and malaise, and quickly progresses to epidermal detachment or skin sloughing and blistering [23]. These skin reactions leave patients at risk of bacteremia and further complications [1,29]. Many cases (80%) involve the eyes, resulting in erythema, discharge, and photophobia [23]. Bronchial, GI, renal, hematologic, and endocrine manifestations are also possible. AHS is an immense concern, and it is imperative to treat the patient promptly if it is suspected following allopurinol treatment.

Pathophysiology of SJS and TEN

SJS/TEN has a complex pathophysiology involving the immune system, genetic predisposition, and a triggering event, including infection or medication exposure. The complexity of this syndrome is not entirely understood but is considered a drug-induced reaction, as over 80-90% of cases of SJS/TEN are caused by medications [30,31]. The population of patients who experience SJS/TEN is not consistent. Thus, there are two mechanisms for this variable drug reaction: immunologic and non-immunologic. A total of 75-80% of adverse drug reactions are secondary to predictable, non-immunologic effects, while 20-25% of adverse drug reactions are caused by unpredictable, immunologic effects [32]. An example of an adverse drug reaction is a drug-induced hypersensitivity reaction. SJS/TEN is a delayed-response hypersensitivity reaction that occurs over 72 hours after exposure to the medication and is T-cell-mediated [31,33]. Many medications are associated with SJS/TEN, including anticonvulsants, allopurinol, antibiotics, and NSAIDs [34]. This rare and unpredictable event contributes to complexity and can result in death [30].

Role of the immune system

When a patient is exposed to a certain infection or medication, immune system mediators activate an immune response consisting of cell damage, apoptosis, and amplification of this process. CD8+ cytotoxic T lymphocytes are the drivers of this immune reaction, as their production is triggered by the incompatibility of the tissue, drug, or infection. CD8+ T cells travel to the skin and stimulate the destruction of cells [31]. Three theories hypothesize how this abnormal T cell response is exaggerated: the Hapten/Pro-Hapten Theory, in which the drug particle and self-protein react to produce a covalently bonded complex recognized at the cell surface; the P-I-Concept, involving a noncovalent interaction of the pharmacological agent and major histocompatibility complex (MHC) immune receptor triggering a T cell response; and the Altered Peptide Theory, in which the triggering agent binds noncovalently to MHC molecules with extreme specificity, changing the shape and chemistry of the molecule to induce T cell stimulation [31,35]. Although these models differ in the interaction between the triggering agent and self-molecules, all theories agree that SJS begins with the activation of T cells. T cells activate an apoptotic cascade involving natural killer cells, cytokines, such as TNF-alpha and interferon-gamma, and other immune mediators that result in necrotic cutaneous and mucosal manifestations recognized as SJS/TEN [36,37]. A study conducted by Kinoshita et al. investigated the role of neutrophils in the blister fluid of SJS/TEN patients and found that neutrophils act as the first responders during this immune response and induce inflammation with the formation of neutrophil extracellular traps in the epidermis. The initiated cascade not only causes inflammation in the skin but also triggers keratinocyte necroptosis [38]. The keratinocyte necroptotic cascade is induced by the interaction between Annexin A1 and formyl peptide receptor 1 [35]. In addition to neutrophils, other mediators that play a role in keratinocyte apoptosis include granulysin and Fas-Fas ligand, which target the bonds between the epidermis and dermis, causing separation, blistering, and sloughing of the skin; both of these mediators were found in high volumes of blister fluid [31]. Perforin and granzyme are released by cytotoxic CD8+ T cells and cause further epidermal damage and detachment of the skin and mucous membranes [35,39].

Role of genetic predisposition

Genetic factors, including specific genetic markers like HLA, put patients at risk for SJS/TEN. HLA alleles present antigens to T cells, resulting in the activation of the immune response [35]. There are certain ethnicities associated with HLA allotypes that have a higher risk of developing SJS/TEN when taking certain medications, including anticonvulsants and allopurinol. For example, in 2004, Chung et al. found that patients of Han Chinese ethnicity with the HLA allotype HLA-B*1502 who take anticonvulsants such as carbamazepine have an increased risk of developing SJS. Specifically, 44 of the 44 patients included in this study who developed SJS or TEN while on carbamazepine displayed this allotype [40]. This association was further examined by investigating other anticonvulsants in the development of SJS and TEN. Phenytoin, lamotrigine, and oxcarbazepine also displayed strong relationships with the development of SJS and TEN [41-43]. In addition, Han Chinese and those of Korean or Thai descent with the HLA allotype HLA-B*5801 who take allopurinol also have the risk of developing SJS/TEN [12,30,35]. HLA allotype screening may be of clinical importance, as ethnic patients can avoid medication exposure if they are at a higher risk of developing SJS/TEN [35]. According to the American College of Rheumatology, allopurinol is contraindicated if Han Chinese patients or patients of Thai descent screen positive for this allotype, along with those of Korean descent with CKD stage 3 [12]. Variability in drug metabolism involving CYP may also play a role in the risk of developing SJS/TEN, as patients who have a slower drug metabolism related to CYP variants can develop adverse drug reactions [35]. Other predispositions that increase the risk of a patient developing SJS/TEN include an immunocompromised state, as well as pediatric patients under the age of 10 and geriatric patients above the age of 80. There has also been a higher incidence of SJS/TEN among women as compared to men [31].

Sample of clinical cases of allopurinol-induced SJS and TEN

Case 1

In a case presented by Wang et al., a 33-year-old male presented to the ED with a one-day history of fever and rash after being unsuccessfully treated for viral prodromal symptoms with amoxicillin and NSAIDs [44]. The patient began taking allopurinol after being diagnosed with gout ten days before the start of his symptoms. The patient had macules with blisters and spontaneous detachment of large sheets of necrolytic epidermis over 60% of his body. In addition, he had mucous membrane involvement with erosions on the lips, oropharynx, genitalia, esophagus, and gastrointestinal tract. The treatment plan included supportive treatment, IV immunoglobulin (IVIg), IV methylprednisolone, and levofloxacin lactate. Reepithelization of the skin was achieved after three weeks. The patient tested positive for HLA-B*58:01, which indicated allopurinol-induced TEN. Patients with the HLA-B*5801 allele have an 80-97 times higher risk of developing allopurinol-induced SJS/TEN than those without the allele [45]. A total of 20% of the Han Chinese population is positive for the allele, whereas 0.7% and 3.8% of Caucasians and African Americans tested positive, respectively [46,47]. However, the HLA-B*5801 allele is not required for allopurinol-induced SJS/TEN, which seems to have multifactorial causes [48].

Case 2

In a case presented by Anis and Meher, a 95-year-old Asian female with a history of CKD, diastolic congestive heart failure, gout, hyperlipidemia, and hypertension presented to the ED with a desquamating rash. She had oral, optic, and genital involvement. The patient had been started on allopurinol ten days before the rash appeared [29]. The patient also had a history of reactions to allopurinol five years ago. Besides her ethnicity, the patient had many of the strong risk factors for allopurinol-induced hypersensitivity reactions identified earlier, including age above 65 years, female sex, previous history of allopurinol-induced reactions, and an eGFR <60 mL/min/1.73 m^2^ (the patient had an eGFR of 15 mL/min/1.73 m^2^) [49]. While hospitalized, her SCORe of TEN was determined to be 3, which represented a 35.3% mortality risk. The patient was treated with IV methylprednisolone, erythromycin 0.5% ointment, ciprofloxacin 0.3%, prednisolone acetate 1%, and cyclosporine 0.05% eye drops for ocular involvement. While hospitalized, the patient developed methicillin-resistant *Staphylococcus aureus* cellulitis. She also developed an acute kidney injury that required hemodialysis while hospitalized. Her skin lesions healed completely, but the patient was ultimately transferred to comfort care with hospice.

Case 3

In a case presented by Gupta et al., an 85-year-old Chinese female presented to the ED with a two-day history of diffuse pruritic maculopapular rash [50]. Eight days prior to her presentation to the ED, she began taking allopurinol; four days after starting the medication, she began having viral prodromal symptoms (fever, sore throat, myalgias, productive cough, and dysphagia). The rash first appeared on her trunk and back, spreading to her hands and feet. She also developed oral and ocular manifestations of the disease, causing hemorrhagic blisters and an inability to open the eyes. Skin biopsy confirmed the TEN diagnosis due to perivascular infiltration of mononuclear cells, epidermal apoptosis, and keratinocyte necrosis. While in the hospital, she went into septic shock. She was treated with IV hydration, empiric antibiotics, dialysis for anuria and acidosis, and surgical debridement of the eyes. The patient continued to need hemodialysis for renal injury, but her rashes improved gradually, and her eyesight was preserved.

Treatment of SJS and TEN

Due to the relative rarity of presentation and the serious nature of SJS and TEN, it is important to consider many factors when deciding on a course of treatment. Of utmost importance is the identification and immediate cessation of the causative agent, as this increases the chances of successful outcomes [51]. Further treatment options are summarized in Table 1 and Table 2 and can only be considered upon successful cessation of the offending drug. Following this crucial step, supportive care has been the mainstay of treatment, although treatment plans seem to vary from institution to institution [52]. It is important to establish a protocol for rapid and effective treatment of SJS, as prompt action is imperative in preventing disease progression.

**Table 1:** Considerations for acute management of SJS/TEN IVIg, intravenous immunoglobulin; SJS, Stevens-Johnson syndrome; TEN, toxic epidermal necrolysis; TNF-alpha, tumor necrosis factor-alpha

Acute management	
Non-pharmacological	Pharmacological
Discontinue the offending agent	Steroids (first line)
Vital signs	Cyclosporine
Maintain the temperature at 30-32 °C	IVIg +/- plasmapheresis
Fluid replacement to maintain a stable urine output of 1 mL/kg/hr	Cyclophosphamide
Maintain an aseptic environment and practice prophylaxis against infection	N-acetylcysteine
Hemodynamic monitoring	IL-6 inhibitors
Nutrient supplementation	TNF-alpha inhibitors
Analgesia	
Specific organ system care	

**Table 2:** Considerations for chronic management of SJS/TEN * Consultation is dependent on the involvement of specific organ systems and mucosal surfaces. SJS, Stevens-Johnson syndrome; TEN, toxic epidermal necrolysis

Chronic management*
Ophthalmologic
Gynecologic
Nephrological
Gastrointestinal/hepatic
Pulmonological

Acute Treatment

Many different agents can be utilized in the treatment of SJS/TEN. There is some debate as to whether it is beneficial to treat patients in burn wards or intensive care units, with some research pointing to favorable outcomes in one area over another, while others show no evidence of improved outcomes regardless of location [53]. Guidelines in the United Kingdom recommend admitting SJS/TEN patients to a burn ward or ICU [54]. Over half of SJS/TEN patients require enteral feeding through a nasogastric tube, mainly depending on the extent of the disease and if patients can adequately meet their nutritional needs via the extent of oral pain and dysphagia associated with mucosal involvement [55].

Non-Pharmacological Treatments

In the event of SJS/TEN with limited resources, it is useful to know there are ways to treat this condition without pharmacological intervention. Before treatment can begin, an assessment of the patient’s vital signs is a necessary step in the management of SJS/TEN [1]. First, maintenance of room temperature in the 30-32 °C range and fluid replacement with a mixture of electrolytes and albumin are indicated to prevent disease progression [53]. Fluid replacement is titrated to maintain a stable urine output of around 1 mL/kg/hour [56]. Maintenance of an aseptic environment is crucial for preventing the spread of infection, which may progress to sepsis [53]. Additional measures that can be taken include continued hemodynamic monitoring, nutrient supplementation, analgesia, and care of skin lesions, mucous membranes, and the eyes [1].

In rural India, wrapping the affected areas in banana leaves has been associated with quicker re-epithelialization and lower pain levels, although care must be taken to avoid infection spreading via the leaves [57]. This can be achieved using banana leaves that have been autoclaved and handled safely. Affected areas are preferentially dressed using paraffin gauze or, more recently, biological membranes, which prevent infection spread and reduce scar formation [53].

Pharmacological Treatments

Steroids have been the mainstay of treatment, with studies showing improved survival rates, potentially longer hospital stays, and increased complications [58-60]. The immunosuppressant effects of steroids are considered the main mechanism of action for their beneficial effects, as this prevents the immunological functions of the cytotoxic T cells and macrophages involved in skin sloughing [61]. Cyclosporine is a commonly used immunomodulator aimed at halting disease progression rather than symptomatic treatment [62]. Cyclosporine treatment prevents further skin sloughing without any increased mortality risk [63]. Additional study is needed to quantify the benefit of tacrolimus, another immunomodulator with a similar mechanism of action to cyclosporine [64]. IVIg has commonly been implemented as a treatment option and has been found to be most effective when coadministered with steroids rather than monotherapy [65]. Other pharmacological interventions include cyclophosphamide, plasmapheresis, IV N-acetylcysteine (reduces the time of re-epithelialization), and biologics aimed at inhibiting specific cytokines [66,67]. IL-6 and TNF-alpha are targets for treatment, as these cytokines are present in higher amounts in SJS/TEN patients [53].

Chronic Treatment

While SJS/TEN is considered mainly a disease of dermatologic manifestation, there is potential involvement of multiple other organ systems once initial treatment has begun. It is recommended that patients almost immediately receive a consult or an examination by an ophthalmologist, as sloughing is particularly detrimental to the eyes, and about three-quarters of SJS/TEN patients develop ocular complications that may even progress to irreversible vision loss [68]. Even with many SJS/TEN patients having ocular sequelae, only about two-thirds of treating physicians consult ophthalmology for their patients [68]. Women should receive a gynecologic exam, as vaginal manifestations are common both acutely and chronically. It is estimated that almost 80% of female SJS/TEN patients have vaginal complications, but these patients only receive a consult or examination about 15% of the time [68]. Other organ systems are commonly affected by epithelial sloughing, which can affect the respiratory, hepatic, renal, and gastrointestinal systems, among others.

## Conclusions

Consideration of the drug allopurinol as a cause for the development of SJS/TEN is of utmost importance, with immediate cessation required in the setting of initial signs and symptoms of the potentially life-threatening condition. While the development of SJS/TEN may be rare, the use of allopurinol in the United States and the surrounding world is extremely prevalent. Thus, providers must be aware of this particular adverse effect of the drug. In this review, we have discussed the mechanism by which allopurinol acts, the treatment indications, especially for the use of gout, and other possible adverse effects of the drug. In addition, the process by which the development of SJS/TEN occurs and the treatment recommendations for SJS/TEN were discussed. Management and treatment options for acute (pharmacologic and non-pharmacologic) and chronic care of SJS/TEN are also described. In order to relate these two topics, three example cases of allopurinol-induced SJS/TEN were also presented. While only three cases were included in the present investigation, it is highly likely that allopurinol is implicated in many more SJS/TEN cases worldwide and should be regarded as the most common drug associated with the development of SJS/TEN.

## References

[1] Zimmerman D, Dang NH (2020). Stevens-Johnson syndrome (SJS) and toxic epidermal necrolysis (TEN): immunologic reactions. Oncologic Critical Care.

[2] Schwartz RA, McDonough PH, Lee BW (2013). Toxic epidermal necrolysis: Part I. Introduction, history, classification, clinical features, systemic manifestations, etiology, and immunopathogenesis. J Am Acad Dermatol.

[3] Schwartz J, Padmanabhan A, Aqui N (2016). Guidelines on the use of therapeutic apheresis in clinical practice-evidence-based approach from the writing committee of the American Society for Apheresis: The Seventh Special Issue. J Clin Apher.

[4] Valeyrie Allanore L, Roujeau JC (2012). Epidermal necrolysis (Stevens-Johnson syndrome and toxic epidermal necrolysis). Fitzpatrick’s Dermatology in General Medicine.

[5] Wolff K, Johnson RA, Saavedra AP, Roh EK (2017). The acutely ill and hospitalized patient. Fitzpatrick’s Color Atlas and Synopsis of Clinical Dermatology.

[6] Koutlas IG (2017). Diseases of the oral cavity. Clinical Dermatology.

[7] Paquet P, Nikkels A, Arrese JE, Vanderkelen A, Piérard GE (1994). Macrophages and tumor necrosis factor alpha in toxic epidermal necrolysis. Arch Dermatol.

[8] Bastuji-Garin S, Fouchard N, Bertocchi M, Roujeau JC, Revuz J, Wolkenstein P (2000). SCORTEN: a severity-of-illness score for toxic epidermal necrolysis. J Invest Dermatol.

[9] Micheletti RG, Rosenbach M, Wintroub BU, Shinkai K (2022). Cutaneous drug reactions. Harrison’s Principles of Internal Medicine.

[10] Smith CF (2017). Erythema multiforme, Stevens-Johnson syndrome, toxic epidermal necrolysis, staphylococcal scalded skin syndrome. Clinical Dermatology.

[11] Yamane Y, Matsukura S, Watanabe Y (2016). Retrospective analysis of Stevens-Johnson syndrome and toxic epidermal necrolysis in 87 Japanese patients - treatment and outcome. Allergol Int.

[12] Qurie A, Preuss CV, Musa R (2024). Allopurinol. StatPearls [Internet].

[13] Stamp LK, O'Donnell JL, Zhang M, James J, Frampton C, Barclay ML, Chapman PT (2011). Using allopurinol above the dose based on creatinine clearance is effective and safe in patients with chronic gout, including those with renal impairment. Arthritis Rheum.

[14] Hoyer D, Atti C, Nuding S, Vogt A, Sedding DG, Schott A (2021). Toxic epidermal necrolysis caused by allopurinol: a serious but still underestimated adverse reaction. Am J Case Rep.

[15] Lavu A, Thiriveedi S, Thomas L, Khera K, Saravu K, Rao M (2021). Clinical utility of HLA-B*58:01 genotyping to prevent allopurinol-induced SJS/TEN. Hosp Pharm.

[16] Khanna D, Khanna PP, Fitzgerald JD (2012). 2012 American College of Rheumatology guidelines for management of gout. Part 2: therapy and antiinflammatory prophylaxis of acute gouty arthritis. Arthritis Care Res (Hoboken).

[17] Fontana RJ, Li YJ, Phillips E, Saeed N, Barnhart H, Kleiner D, Hoofnagle J (2024). Allopurinol hepatotoxicity is associated with human leukocyte antigen Class I alleles. Liver Int.

[18] Choi W, Yang YS, Chang DJ (2021). Association between the use of allopurinol and risk of increased thyroid-stimulating hormone level. Sci Rep.

[19] Abhishek A, Roddy E, Doherty M (2017). Gout - a guide for the general and acute physicians. Clin Med (Lond).

[20] Engel B, Just J, Bleckwenn M, Weckbecker K (2017). Treatment options for gout. Dtsch Arztebl Int.

[21] (2023). Evidence reviews for timing of urate-lowering therapy in relation to a flare in people with gout: Gout: diagnosis and management. https://pubmed.ncbi.nlm.nih.gov/36063469/.

[22] FitzGerald JD, Dalbeth N, Mikuls T (2020). 2020 American College of Rheumatology guideline for the management of gout. Arthritis Care Res (Hoboken).

[23] Turnheim K, Krivanek P, Oberbauer R (1999). Pharmacokinetics and pharmacodynamics of allopurinol in elderly and young subjects. Br J Clin Pharmacol.

[24] Day RO, Graham GG, Hicks M, McLachlan AJ, Stocker SL, Williams KM (2007). Clinical pharmacokinetics and pharmacodynamics of allopurinol and oxypurinol. Clin Pharmacokinet.

[25] Ryu HJ, Song R, Kim HW (2013). Clinical risk factors for adverse events in allopurinol users. J Clin Pharmacol.

[26] Stamp LK, Day RO, Yun J (2016). Allopurinol hypersensitivity: investigating the cause and minimizing the risk. Nat Rev Rheumatol.

[27] Young JL Jr, Boswell RB, Nies AS (1974). Severe allopurinol hypersensitivity. Association with thiazides and prior renal compromise. Arch Intern Med.

[28] Lonjou C, Borot N, Sekula P (2008). A European study of HLA-B in Stevens-Johnson syndrome and toxic epidermal necrolysis related to five high-risk drugs. Pharmacogenet Genomics.

[29] Anis TR, Meher J (2023). Allopurinol-induced Stevens-Johnson Syndrome (SJS). Clin Pharmacol.

[30] Oakley AM, Krishnamurthy K (2024). Stevens-Johnson syndrome. StatPearls [Internet].

[31] Hanson LM, Bettencourt AP (2020). Stevens-Johnson syndrome and toxic epidermal necrolysis: a guide for nurses. AACN Adv Crit Care.

[32] Al Aboud DM, Nessel TA, Hafsi W Cutaneous adverse drug reaction. StatPearls [Internet].

[33] Cheng L (2021). Current pharmacogenetic perspective on Stevens-Johnson syndrome and toxic epidermal necrolysis. Front Pharmacol.

[34] Lee EY, Knox C, Phillips EJ (2023). Worldwide prevalence of antibiotic-associated Stevens-Johnson syndrome and toxic epidermal necrolysis: a systematic review and meta-analysis. JAMA Dermatol.

[35] Hasegawa A, Abe R (2020). Recent advances in managing and understanding Stevens-Johnson syndrome and toxic epidermal necrolysis. F1000Res.

[36] Arnold KA, Gao J, Stein SL (2019). A review of cutaneous hypersensitivity reactions in infants: from common to concerning. Pediatr Dermatol.

[37] Hsieh MH, Watanabe T, Aihara M (2021). Recent dermatological treatments for Stevens-Johnson syndrome and toxic epidermal necrolysis in Japan. Front Med (Lausanne).

[38] Kinoshita M, Ogawa Y, Hama N (2021). Neutrophils initiate and exacerbate Stevens-Johnson syndrome and toxic epidermal necrolysis. Sci Transl Med.

[39] Liotti L, Caimmi S, Bottau P (2019). Clinical features, outcomes and treatment in children with drug induced Stevens-Johnson syndrome and toxic epidermal necrolysis. Acta Biomed.

[40] Chung WH, Hung SI, Hong HS (2004). A marker for Stevens-Johnson syndrome. Nature.

[41] Locharernkul C, Loplumlert J, Limotai C (2008). Carbamazepine and phenytoin induced Stevens-Johnson syndrome is associated with HLA-B*1502 allele in Thai population. Epilepsia.

[42] Chen CB, Hsiao YH, Wu T (2017). Risk and association of HLA with oxcarbazepine-induced cutaneous adverse reactions in Asians. Neurology.

[43] Cheung YK, Cheng SH, Chan EJ, Lo SV, Ng MH, Kwan P (2013). HLA-B alleles associated with severe cutaneous reactions to antiepileptic drugs in Han Chinese. Epilepsia.

[44] Wang F, Ma Z, Wu X, Liu L (2019). Allopurinol-induced toxic epidermal necrolysis featuring almost 60% skin detachment. Medicine (Baltimore).

[45] Somkrua R, Eickman EE, Saokaew S, Lohitnavy M, Chaiyakunapruk N (2011). Association of HLA-B*5801 allele and allopurinol-induced Stevens Johnson syndrome and toxic epidermal necrolysis: a systematic review and meta-analysis. BMC Med Genet.

[46] Ko TM, Tsai CY, Chen SY (2015). Use of HLA-B*58:01 genotyping to prevent allopurinol induced severe cutaneous adverse reactions in Taiwan: national prospective cohort study. BMJ.

[47] Jutkowitz E, Dubreuil M, Lu N, Kuntz KM, Choi HK (2017). The cost-effectiveness of HLA-B*5801 screening to guide initial urate-lowering therapy for gout in the United States. Semin Arthritis Rheum.

[48] Kang HR, Jee YK, Kim YS (2011). Positive and negative associations of HLA class I alleles with allopurinol-induced SCARs in Koreans. Pharmacogenet Genomics.

[49] Do MD, Mai TP, Do AD (2020). Risk factors for cutaneous reactions to allopurinol in Kinh Vietnamese: results from a case-control study. Arthritis Res Ther.

[50] Gupta SS, Sabharwal N, Patti R, Kupfer Y (2019). Allopurinol-induced Stevens-Johnson syndrome. Am J Med Sci.

[51] Chang HC, Wang TJ, Lin MH, Chen TJ (2022). A review of the systemic treatment of Stevens-Johnson syndrome and toxic epidermal necrolysis. Biomedicines.

[52] Langley A, Worley B, Pardo Pardo J (2018). Systemic interventions for treatment of Stevens‐Johnson syndrome (SJS), toxic epidermal necrolysis (TEN), and SJS/TEN overlap syndrome. Cochrane Database Syst Rev.

[53] Kumar R, Das A, Das S (2018). Management of Stevens-Johnson syndrome-toxic epidermal necrolysis: Looking beyond guidelines!. Indian J Dermatol.

[54] Noe MH, Micheletti RG (2020). Diagnosis and management of Stevens-Johnson syndrome/toxic epidermal necrolysis. Clin Dermatol.

[55] McCullough M, Burg M, Lin E, Peng D, Garner W (2017). Steven Johnson syndrome and toxic epidermal necrolysis in a burn unit: a 15-year experience. Burns.

[56] Letko E, Papaliodis DN, Papaliodis GN, Daoud YJ, Ahmed AR, Foster CS (2005). Stevens-Johnson syndrome and toxic epidermal necrolysis: a review of the literature. Ann Allergy Asthma Immunol.

[57] Srinivas CR, Sundaram VS, Raju BA, Prabhu SK, Thirumurthy M, Bhaskar AC (2006). Achieving asepsis of banana leaves for the management of toxic epidermal necrolysis. Indian J Dermatol Venereol Leprol.

[58] Gerdts B, Vloemans AF, Kreis RW (2007). Toxic epidermal necrolysis: 15 years' experience in a Dutch burns centre. J Eur Acad Dermatol Venereol.

[59] Chave TA, Mortimer NJ, Sladden MJ, Hall AP, Hutchinson PE (2005). Toxic epidermal necrolysis: current evidence, practical management and future directions. Br J Dermatol.

[60] Lee HY, Dunant A, Sekula P (2012). The role of prior corticosteroid use on the clinical course of Stevens-Johnson syndrome and toxic epidermal necrolysis: a case-control analysis of patients selected from the multinational EuroSCAR and RegiSCAR studies. Br J Dermatol.

[61] Michaels B (2009). The role of systemic corticosteroid therapy in erythema multiforme major and stevens-johnson syndrome: a review of past and current opinions. J Clin Aesthet Dermatol.

[62] Singh GK, Chatterjee M, Verma R (2013). Cyclosporine in Stevens Johnson syndrome and toxic epidermal necrolysis and retrospective comparison with systemic corticosteroid. Indian J Dermatol Venereol Leprol.

[63] Valeyrie-Allanore L, Wolkenstein P, Brochard L (2010). Open trial of ciclosporin treatment for Stevens-Johnson syndrome and toxic epidermal necrolysis. Br J Dermatol.

[64] Dogra PM, Chatterjee M, Neema S (2015). Tacrolimus for treatment of toxic epidermal necrolysis. Indian J Dermatol Venereol Leprol.

[65] Shanbhag SS, Chodosh J, Fathy C, Goverman J, Mitchell C, Saeed HN (2020). Multidisciplinary care in Stevens-Johnson syndrome. Ther Adv Chronic Dis.

[66] Jagadeesan S, Sobhanakumari K, Sadanandan SM, Ravindran S, Divakaran MV, Skaria L, Kurien G (2013). Low dose intravenous immunoglobulins and steroids in toxic epidermal necrolysis: a prospective comparative open-labelled study of 36 cases. Indian J Dermatol Venereol Leprol.

[67] Frangogiannis NG, Boridy I, Mazhar M, Mathews R, Gangopadhyay S, Cate T (1996). Cyclophosphamide in the treatment of toxic epidermal necrolysis. South Med J.

[68] Hasan MJ, Rabbani R (2020). Intravenous N-acetylcysteine in severe cutaneous drug reaction treatment: a case series. SAGE Open Med Case Rep.

